# The Effect of Photodynamic Therapy and Diode Laser as Adjunctive Periodontal Therapy on the Inflammatory Mediators Levels in Gingival Crevicular Fluid and Clinical Periodontal Status

**Published:** 2016-09

**Authors:** Faraz Teymouri, Shirin Zahra Farhad, Hedayatollah Golestaneh

**Affiliations:** 1 Postgraduate Student, Dept. of Periodontics, School of Dentistry, Isfahan (Khorasgan) Branch, Islamic Azad university, Isfahan, Iran.; 2 Dept. of Periodontics, School of Dentistry, Isfahan (Khorasgan) Branch, Islamic Azad University, Isfahan, Iran.

**Keywords:** Cytokines, Laser Therapy, Photosan

## Abstract

**Statement of the Problem:**

The presence of bacterial biofilms is the major cause of gingivitis and periodontitis, their mechanical removal is not often enough. Therefore, laser therapy and photodynamic therapy can be effective as adjunctive treatment.

**Purpose:**

This study aimed to evaluate the impact of these treatments on the level of gingival crevicular fluid (GCF), inflammatory mediators, and periodontal clinical status.

**Materials and Method:**

In this clinical trial, three quadrants were studied in 12 patients with chronic periodontitis aged 30-60 years. The clinical parameters were recorded and GCF samples were taken. After the first phase of periodontal treatment, one of the three quadrants was determined as the control group, one was treated by diode laser, and one underwent photodynamic therapy. The clinical parameters were recorded 2 and 6 weeks later. The data were statistically analyzed by using Friedman, ANOVA, and LSD post-test.

**Results:**

Significant reduction was observed over time in the level of Interleukin-1β (IL-1β), Interleukin-17 (IL-17), clinical attachment loss, and pocket depth in the three treatment groups (*p*< 0.000). The three treatment methods significantly reduced the IL-1β and IL-17 at the baseline, up to 2 weeks, and 2-6 weeks (*p*< 0.05). Diode laser and photodynamic therapy significantly decreased the average bleeding on probing over time (*p*< 0.000 and *p*< 0.002, respectively).

**Conclusion:**

Laser and photodynamic therapy reduced the inflammatory mediators (IL-1β and IL-17) and improved the clinical symptoms.

## Introduction

Periodontitis is an inflammatory disease of the tooth supporting tissues which is caused by a group of specific microorganisms. It vastly destroys periodontal ligament and alveolar bone, along with pocket formation and/or gingival recession.[1]

Treatment of all cases of periodontal diseases includes mechanical cleaning of the tooth surfaces to remove the mineralized and non-mineralized bacterial deposits (germ and plaque).[2] The presence of bacterial biofilms containing periodontal pathogens on tooth surface is considered as the main cause of gingivitis and periodontitis.[3]

In chronic infections of connective tissue, adhesion and invasion of bacterial pathogens to the host tissues can trigger the destruction of structural proteins. Besides, over-production of inflammatory mediators including Interleukin-1β (IL-1β) and Interleukin-17 (IL-17) by the host cells such as fibroblasts, other periodontal cells, and infiltrated induced cells at the site plays a role in the destruction of periodontal tissues.[4-5] New methods for treatment of periodontitis target the etiologic factors to eliminate the chain of reactions caused by bacteria in the human immune system, because these reactions lead to the release of inflammatory mediators that are responsible for the destruction of connective tissue and bone. Thus, the basic treatment involves removal of both local and systemic factors.[6] Long-term studies have reported substantial decrease in gingival crevicular fluid (GCF) inflammatory mediator levels. Considering the problems and side effects of chemical agents which are used to influence these mediators, a new set of practices such as laser therapy or photodynamic therapy (PDT) seems necessary as adjunctive periodontal treatment. Laser penetrates into the tissues and body fluids and selectively affects the inflammatory mediators.

PDT, with fewer side effects, is another alternative to chemical antimicrobial agents for removal of subgingival bacteria and treatment of periodontitis.[7]

The clinical implications of these new treatments have been partly studied and contradictory results were obtained. Few studies extendedly examined the localized effects of these methods on inflammatory mediators associated with periodontal diseases in human, and proved the role of these mediators in innate and acquired immunity in periodontitis. They suggested that analysis of the mediators in biological fluids of the body can help determining the current status of periodontal destruction.[8]

Garcia *et al.*[10] found that employing PDT for plaque removal and root surface planing reduced the inflammatory mediators.[9] In another study, Makhlouf *et al.* reported that the reduction of gingival index, plaque index, and IL-1β level in GCF was not significant in the group treated with laser.

De Micheli *et al.*[11] evaluated the effects of antibacterial agents and diode laser on the periodontal clinical parameters and observed no clinical or microbiological difference between the experimental and control group. Giannelli *et al.* detected that laser therapy significantly decreased the rate of periodontal pathogens.[12]

The limited number of previous studies and their inconsistent results had us design a study to evaluate and compare the impact of laser and photodynamic therapy as complementary periodontal treatments on the clinical status and levels of inflammatory mediators as new mechanisms for evaluating periodontal diseases, especially the new mediators. 

## Materials and Method

The current clinical trial was registered at the Iranian Registry of Clinical Trials (#IRCT2014040617145N1). Twelve patients with moderate to severe generalized chronic periodontitis were selected out of those referred to the Periodontics ward at the School of Dentistry, Azad University of Isfahan. The patients were aged 30-60 years old and had at least four sites with probing depth of 4-6 mm in each quadrant. The exclusion criteria were pregnancy and lactation, certain systemic diseases, using specific medications, drugs or alcohol, systemic use of antibiotics over the preceding 6 months, having received periodontal treatment within the last year, and plaque index of >40%.

Informed consents were obtained, the preliminary examinations were done, and periodontal files were completed for each patient. Then, the clinical parameters including bleeding on probing (BoP), probing depth (PD), and clinical attachment loss (CAL) were measured and recorded. After isolating the area in each quadrant, GCF samples were taken from 4 areas of the deepest pocket by using paper points NO.25 and were placed in a special transport medium. The samples were all placed on ice in special containers at 2-5° C, and were transferred to the laboratory in less than 30 minutes to be kept at a temperature of -70° C.

In the diode laser treatment quadrant, after GCF sampling, the first phase of laser treatment (initial laser curettage) was carried out by using 810 nm laser diode set (UNIVENT; Italy) only to reduce the number of periodontal bacteria. Then, the first stage of****scaling and root planing (SRP) was done by using ultrasonic device.

In photodynamic therapy, a light source exposes the photosensitizer to a specific wave-length of low-power visible light. A 630-700 nm red light is enough to activate most photosensitizers, with 0.5-1.5 cm light penetration depth which restricts the depth of necrosis. The total light dose, dose rates, and the depth of destruction vary with each tissue treated and photosensitizer used. The light sources being currently employed in PDT are helium–neon lasers (633 nm), gallium–aluminum–arsenide diode lasers (630-690, 830 or 906 nm), and argon laser (488-514 nm) with a range of wavelength from visible light to the blue of argon lasers, or from the red of helium-neon laser to the infrared area of diode lasers. Non-laser light sources such as light-emitting diodes (LED) have been recently employed as new light activators in PDT. Compared with conventional lasers, LED devices are more compact, portable, and cost-effective.[13]

In the control quadrant, the first phase of periodontal treatment was performed. In the PDT quadrant, the treatment procedure was performed by using Photosan system (DK-2800; CMS Dental, Copenhagen, Denmark) and toluidine blue as the light-sensitive material. The material was inserted into the dental pockets and irradiated by periodontal head no.15 for 10 seconds. Then, the first phase of the SRP was done by using ultrasonic device.

After a week of initial treatment, the second phase of laser treatment (laser-assisted periodontal therapy) was performed. In this step, the de-epithelization of sulcus depth was done to induce delayed re-growth of the epithelium. This procedure was also done on the outer surface of the gingival margin and inside the sulcus. Then, the second phase of SRP was carried out on the three quadrants with ultrasonic instruments and ended with hand instruments.

Finally, the patients were recalled 2 and 6 weeks after the first treatment session. The clinical parameters were recorded and the GCF samples were recollected. The samples taken *in vitro* were incubated in special media and were analyzed by special ELISA kit and Elisa Reader device. The obtained data were statistically analyzed by using SPSS software, version 18. The Friedman test was performed to compare the means of the groups. ANOVA was used for multiple group comparisons at different times, and LSD post-test was performed to compare the two groups. Moreover, *p*< 0.05 was considered as significance level.

## Results

This study was conducted on three groups with different treatments including laser treatment, photodynamic therapy, and the control group. The observations and statistical tests revealed that the mean values of IL-1β and IL-17 reduced significantly in the laser treatment (*p*< 0.000), PDT (*p*< 0.000) and control group (*p*< 0.007) at the baseline, 2 weeks and 6 weeks after the treatment. (Table 1)

**Table 1:** The mean±SD of IL-1β and IL-17 at different times

		**IL-1β**	**IL-17**	**p-value**
		**Mean±SD**	**Mean±SD**	
Laser	Initial 2^nd^ week 6^th^ week	16 ±3.17 10 ± 3.70 5 ± 2.38	9 ± 3.54 5 ± 2.50 2.4 ± 1.52	0.000
PDT	Initial 2^nd^ week 6^th^ week	21 ± 4.98 17 ± 4.87 12 ± 4.21	9.8 ± 3 7.5 ± 3 5.2 ± 2	0.000
Control	Initial 2^nd^ week 6^th^ week	15 ± 3.95 8 ± 4.56 10 ± 5.28	5.4 ± 2 2.2 ± 0.77 3.1 ± 0.72	0.007

The mean baseline and 2-week values of IL-1β were not significantly different in the study groups (*p*= 0.142). However, comparing the baseline and the 6-week values revealed a significant decrease after the treatment (*p*< 0.002), and from the 2^nd^ to the 6^th^ week (*p*< 0.048). (Table 2)

**Table 2:** The mean±SD of IL-1β in 2 and 6 weeks after the treatment

		**Mean±SD (ng/ml)**	**p-value**
Initial-2^nd^ week	Laser PDT Control	5.9 ± 2.68 4.4 ± 2.55 7.1 ± 2.37	0.142
Initial-6^th^ week	Laser PDT Control	11 ± 1.81 9 ± 3.23 6 ± 1.76	0.002
2^nd^-6^th^ week	Laser PDT Control	5 ± 2.13 5 ± 3.69 1 ± 2.01	0.05

**Table 3:** The mean±SD of IL-17 at different times after the treatment

		**Mean±SD** **(ng/ml)**	**p-value**
Initial-2^nd^ week	Laser PDT Control	4.0 ± 2.27 2.3 ± 0.75 3.2 ± 1.87	0.065
Initial-6^th^ week	Laser PDT Control	6.5 ± 2.34 4.6 ± 1.33 2.2 ± 1.48	0.001
2^nd^-6^th^ week	Laser PDT Control	2.5 ± 1.30 2.4 ± 1.22 1.0 ± 0.55	0.04

No significant difference was observed between the baseline and 2^nd^ week mean values of IL-17 in the three treatment groups (*p*= 0.065). Nonetheless, the mean values significantly declined from the baseline to the 6^th^ week (*p*< 0.001) and from the 2^nd^ to the 6^th^ week (*p*< 0.004). (Table 3) Significant reduction was found in the mean clinical attachment level and probing depth at the baseline, 2^nd^, and 6^th^ weeks after the treatments in the laser (*p*< 0.000), PDT (*p*< 0.00), and control group (*p*< 0.03). (Table 4)

**Table 4:** Comparison of the mean values of CAL and PD of the study groups at different times

		**IL-1β**	**IL-17**	**p-value**
		**Mean±SD**	**Mean±SD**	
Laser	Initial 2^nd ^week 6^th^ week	3.3 ± 0.19 2.8 ± 0.15 2.4 ± 0.12	4.9 ± 0.99 3.5 ± 0.51 2.7 ± 0.45	0.000
PDT	Initial 2^nd ^week 6^th^ week	3.5 ± 0.22 3.2 ±0.16 2.8 ± 0.18	4.9 ± 0.99 3.7 ± 0.62 2.8 ± 0.38	0.000
Control	Initial 2^nd ^week 6^th^ week	3.4 ± 0.18 3.2 ± 0.12 2.9 ± 0.18	4.8 ± 0.83 4 ± 0.0 3 ± 0.0	0.037

**Figure 1:**
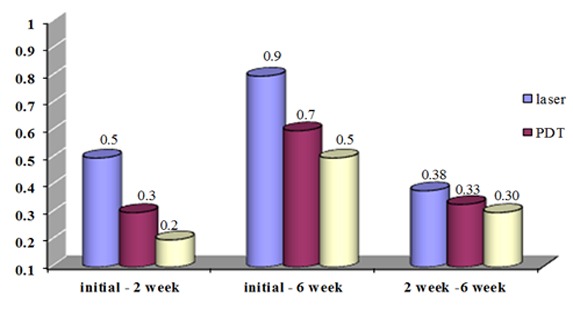
The mean of CAL at different times after the treatment

The mean clinical attachment level of the three treatment groups was not significantly different between the baseline and 2^nd^ week values (*p*= 0.225), the baseline and 6^th^ week values (*p*= 0.295), and the 2^nd^ and 6^th^ week values (*p*= 0.891). (Figure 1)

**Figure 2:**
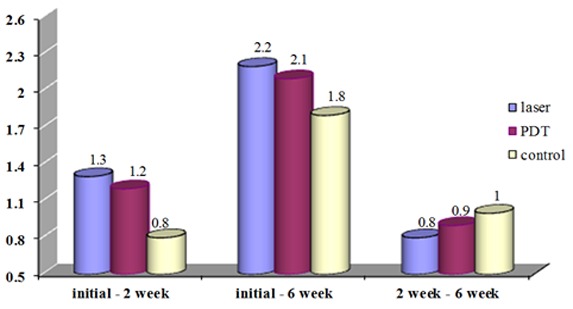
The mean of PD at different times after the treatment

No significant reduction was detected in the mean pocket depth of the three groups between the baseline and the 2^nd^ week (*p*=0.477), the baseline and the 6^th^ week (*p*= 0.750) and the 2^nd^ and 6^th^ week after the treatment (*p*= 0.852). (Figure 2)

The mean BoP reduced significantly at the baseline, 2^nd^ and 6^th^ week in the laser (*p*< 0.000) and PDT groups (*p*< 0.02); however, it showed no significant difference in the control group (*p*= 0.223).

**Figure 3:**
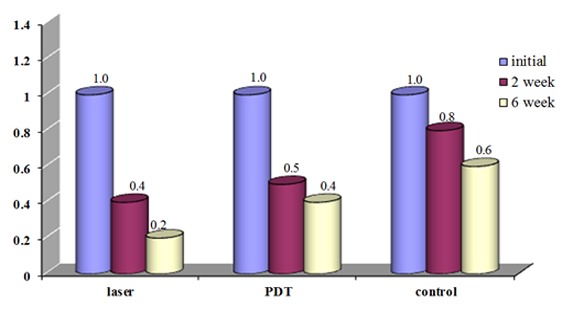
The mean of BoP at different times after the treatment

The mean bleeding on probing in the three treatment groups was not significantly different from the baseline to the 2^nd^ week after the treatment (*p*= 0.363), from the baseline to the 6^th^ week (*p*= 0.194), and from the 2^nd^ to the 6^th^ weeks after the treatment (*p*= 0.560). (Figure 3)

## Discussion

Based on the results obtained from the laser, photodynamic therapy, and control groups, the inflammatory mediators reduced dramatically, which consequently led to improved clinical parameters, namely CAL, PD, and BOP. However, no significant difference was observed in some groups.

The findings indicated a significant reduction in IL-1β levels in the three groups, particularly in the final weeks of the study. The control group was significantly different from the laser group and PDT group; whereas, the laser and PDT groups were not significantly different.

Garcia *et al.*[9] stated in their research that a combined treatment of PDT, low concentrations of methylene blue, and toluidine blue was the most effective adjunctive periodontal treatment associated with scaling and root planning, which indirectly reduced the inflammatory mediators and controlled the bone resorption in periodontitis.

Likewise, Queiroz *et al.*[14] reported that the adjunctive PDT therapy could not completely improve the clinical parameters in smokers; although, it suppressed the IL-1β and MMP-8 compared with SRP per se. Similar results were found by Lui *et al.*[15] and Kolbe *et al.*[16] which were all consistent with the findings of the present study.

Meanwhile, Pourabbas *et al.*[17] reported a significant decrease in IL-1β in the PDT group, which was not in line with our study. Such a difference in results might be due to the various types of dye used in their study and the current one. According to some studies, certain dyes may reduce specific inflammatory mediators, since the dyes reacts with some certain mediators and make them more exposed to the applied radiation; a phenomenon that does not happen with other dye reagents.

Frequency of use is another determining factor for the influence of PDT on inflammatory mediators about which there are conflicting opinions. Malik *et al.*[18] stated in their article that repeating the PDT procedure for several times in the first weeks of treatment would increase the anti-inflammatory effects. However, the single-dose application of PDT in the present study was in agreement with a number of studies such as Andersen *et al.*’s,[19] which dramatically reduced the inflammatory mediators.

Concerning the impact of laser treatment on the inflammatory mediators, the findings showed that the reduction of these mediators in this group was more considerable than that in the PDT and control groups; and that the decrease was significant in the last weeks. Thus, it can be said that as the time passed and the mechanical and adjunctive treatments were repeated, these mediators reduced more and more.

Calderin *et al.*[20] concluded that the laser treatment decreased the IL-1β and TNF-α significantly more than performing only the first phase of treatment; however, repeating the sessions of laser therapy expedited the achievement of the desirable result. Yamaura *et al.*[21] detected that low-level laser therapy decreased the levels of TNF-α, IL-1β, and IL-8. They also observed that the mechanism had positive effects on periodontal inflammatory conditions. These studies were all consistent with the present study.

Generally, it is too difficult to specifically compare the laser therapy and the conventional method of periodontal treatment due to the different laser wavelengths, the vast variety of laser parameters, lack of adequate records to calculate the laser density, and differences in the experimental studies.[22] Moreover, the type of laser, durations of the study and laser therapy, the radiation dose applied, and the frequency of laser treatment can also affect the obtained results. Makhlouf *et al.*[10] revealed that laser treatment caused no significant reduction in gingival index, plaque index, and IL-1β levels in GCF. The applied radiation dose and frequency of laser treatment in Makhlouf’s study was different from our study. Qadri *et al.*[23] observed that the two groups of SRP per se and laser treatment were not significantly different in terms of the reduction of IL-1β. The duration of their study and laser treatment period was longer than the current study.

The present study revealed that the difference between the effect of laser treatment and PDT on the level of IL-1β was not significant; while, the two groups were significantly different regarding their effect on the level of IL-17. It can be justified as the dyes used in PDT method to influence the inflammatory mediators were absorbed differently and selectively by various cells. Hence, the absorption, and thereby, the effect of PDT on IL-17 would possibly be more than the IL-1β, resulting in significant difference between the laser group and PDT group in terms of IL-17 levels.

It must be noted that the radiation dose applied in treatments might be appropriate for a mediator and inappropriate for another at the same time. Giannopoulou *et al.*[2] found no significant difference between the effects of these two treatment methods on IL-1β and IL-17; which contrasts the results of our study. It might be due to the use of a dye different from the reagent used in this study, as well as the longer duration of the study.

Concerning the clinical parameters in this study, it was noted that the PDT improved the clinical symptoms (CAL, PD, and BoP) during the course of treatment (2 and 6 weeks); this improvement was significant in BoP parameter.

In another study, Atieh[24] concluded that combining PDT with SRP would result in a dramatically decreased pocket depth, higher level of attachments, and slightly greater gingival recession. Moreover, the scale of general mouth bleeding declined significantly.

In their study, Christodoulides *et al.*[25] found no statistically significant difference between the two groups in terms of PD and CAL; however, the general oral bleeding index had a significantly greater decrease in the test group. These results were consistent with the current study.

Ruhling *et al.*[26] conducted a study in 2010 and observed that the two groups did not differ significantly in terms of plaque index, BoP, and the probing pocket depth. Consequently, their hypothesis suggesting the preference of PDT over periodontal mechanical treatment was rejected. This difference can be probably attributed to the longer duration of other studies, and the repeated therapeutic doses of PDT used in some studies versus the single-session PDT treatment in our study.

Furthermore, our findings revealed that as in the PDT group, the improvement of clinical parameters in laser-treated samples was higher than the control group; the difference was significant in the case of BoP. The results of this study were in full agreement with Slot *et al.’*s study,[27] in which it was observed that compared with the control group, laser therapy improved PD and CAL moderately, and improved BoP significantly. De Micheli *et al.*[11] stated that adjunctive laser treatment created no significant difference between the study and control groups. This inconsistency between some studies and the current one may be due to the similar reasons mentioned about the PDT effects on parameters.

## Conclusion

Within the conditions of this study, it can be concluded that regarding the anti-inflammatory effects of periodontal treatment methods, adjunctive PDT and laser treatment significantly reduce the level of inflammatory mediators. This impact increases towards the final weeks of the process. These treatments also improve the clinical symptoms, particularly BoP, which is again more efficient when prolonged. 
